# Human health risk analysis from disinfection by-products (DBPs) in drinking and bathing water of some Indian cities

**DOI:** 10.1186/2052-336X-12-73

**Published:** 2014-04-23

**Authors:** Brijesh Kumar Mishra, Sunil Kumar Gupta, Alok Sinha

**Affiliations:** 1 Department of Environmental Science and Engineering, Indian School of Mines, Dhanbad 826004, India

**Keywords:** Disinfection, Drinking water, Exposure assessment & risk

## Abstract

**Background:**

Human health risk assessment from exposure to disinfection by-products (DBPs) during drinking and bathing water vary from country to country as per life expectancy, body mass index, water consumption pattern and individual concentration of DBPs component, etc.

**Methods:**

Present study considered average direct water intake per person for adult males and females as 4 & 3 L/day, respectively as per Indian literature for risk evaluation from another component of pollutant. While other important factor like average life expectancy, body weight & body surface area for male and female were considered 64 & 67 years, 51.9 & 45.4 Kg and 1.54 & 1.38 m^2^ respectively as per Indian Council of Medical Research and WHO report. The corresponding lifetime cancer risk of the formed THMs to human beings was estimated by the USEPA and IRIS method as per Indian population.

**Results:**

The total cancer risk reached 8.99 E-04 and 8.92 E-04 for males and females, respectively, the highest risk from THMs seems to be from the inhalation route followed by ingestion and dermal contacts.

**Conclusions:**

The multipath way evaluations of lifetime cancer risks for THMs exposure through ingestion, dermal absorption, and inhalation exposure were examined at the highest degree of danger. Results reveals that water containing THMs of the selected water treatment plant of the eastern part of India was unsafe in terms of risk evaluation through inhalation and ingestion, while dermal route of risk was found very close to permissible limit of USEPA. Sensitivity analysis shows that every input parameter is sole responsible for total risk potential, whereas exposure duration playing important role for estimation of total risk.

## Introduction

DBPs are produced when disinfectants such as chlorine, ozone, chloramine, or chlorine dioxide react with naturally occurring organic matter in the water. Chlorination is most economical and feasible disinfectant in India, but it generates various chlorine byproducts (CBPs) which are potential carcinogens, especially halogenated organic by-products such as trihalomethanes (THMs). The WHO reported that the highest concentration of chlorine by-products was THMs which consist of four compounds: chloroform (CHCl_3_), bromodichloromethane (CHCl_2_Br), dibromochloromethane (CHClBr_2_), and bromoform (CHBr_3_) [1]. USEPA reported that these four THMs are human carcinogens, of which CHCl_3_, CHCl_2_Br and CHBr_3_ are carcinogen type B2 (probable human carcinogens) and CHClBr2 is carcinogen type C (possible carcinogens) [2]. Epidemiologic studies of chlorinated drinking water exposures in humans suggest weak associations primarily with bladder, rectal, and colon cancer [3-9] and limited evidence of reproductive and developmental effects [10-13]. DBPs risk analysis is used for establishing, explaining, and estimating any substantive human health risks from exposure to DBPs found in drinking water. Drinking water is not used exclusively for drinking but also for cooking, bathing, rinsing, cleaning, etc. As a result, for many drinking water contaminants, there is the potential for exposure and uptake not only by ingestion but also by dermal absorption and inhalation. Traditional risk assessments for water often consider only ingestion exposure to toxic chemicals. Since 1990, scientists proposed that inhalation and dermal absorption be considered in the risk assessment of drinking water, which considered more input parameter like skin surface area, THMs concentration in air, ventilation rate, bathroom volume, air flow rate [14-19]. In 1986, as part of the Safe Drinking Water Amendments, the US Environmental Protection Agency (USEPA) proposed the Disinfectants/DBPs Rule Stage I & II. Under Stage I, the MCL for total THMs was set at 80 mg/L where as in Stage II, the MCL is expected to further decrease to 40 mg/L to reduce the level of risk potential of human health [20,21]. Whereas WHO and Indian standard of drinking water IS 10500 considered the permissible limit of THM for each component as (Chloroform = 300 μg/L; BDCM =60 μg/L; DBCM = 100 μg/L and Bromoform = 100 μg/L) which is higher than the permissible limit of USEPA standard.

The present study is concentrated on to understanding the consequences of the future application of risk assessment from THMs especially when chloroform is more contributor THMs in drinking water. In addition, cancer risk estimation was carried out considering exposure by ingestion, inhalation, and dermal contact. Human health risk assessment from exposure to disinfection by-products (DBPs) during drinking and bathing water varies from country to country as per life expectancy, body mass index and water consumption, etc.

## Materials and methods

### Collection, storage and analysis of water sample

The water samples for THM analysis were collected in 40-mL amber glass vials with polypropylene screw caps and TFE-faced septa and quenched immediately with ascorbic acid (30 mg/l) to eliminate further formation of THMs. The vials were carefully filled so that trapping of air bubbles inside was prevented. All samples were kept in the dark at +4°C until analysis.

### TTHMs analysis

THM analysis was performed by CHEMITO CERES-800 PLUS gas chromatograph by liquid-liquid extraction (LLE) with pentane (EPA Method 551.1) having a detection limit for Chloroform = 0.010 μg/L; BDCM = 0. 005 μg/L; DBCM = 0. 007 μg/L and Bromoform =0. 010 μg/L with a recovery rate for Chloroform = 99; BDCM = 97; DBCM = 98. 3 and Bromoform =94. Separation and quantification of individual THM compounds were analyzed with Ni63 EC while fused silica capillary column DB5, 30 M × 0.5 mm (id) was utilized for the chromatographic separation of individual THMs. Injector and detector temperatures were kept at 200°C and 250°C, respectively. The oven temperature was programmed to remain constant at 40°C for 3 min and rise to 150°C at a ramp rate of 8°C/min. Nitrogen was used as a carrier gas at a flow rate of 60 mL/min.

### Risk analysis

**Figure 1:**
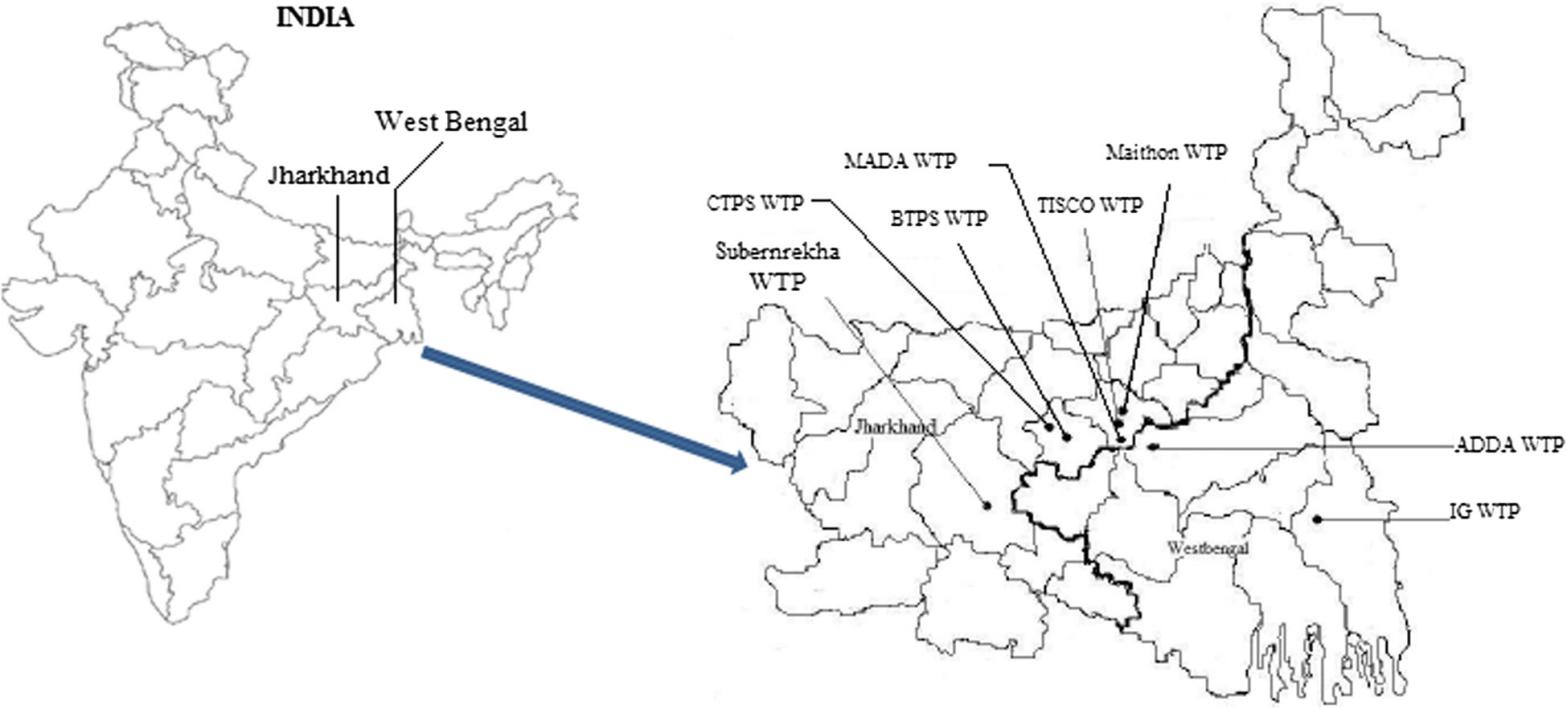
Location of WTP in selected region of Jharkhand and West Bengal.

Human health risk analysis from disinfection by-products (DBPs) in drinking and bathing purpose is based on the data collected from eight different water treatment plant located in Jharkhand and West Bengal namely BTPS water treatment plant, MADA water treatment plant, CTPS water treatment plant, Maithon water treatment plant, Indira Gandhi water treatment plant, TISCO water treatment plant, ADDA water treatment plant and Swarnrekha water treatment plant during the period 2012–13 as Figure 1. Present study explored the exposure assessment from major carcinogenic THMs in terms of Chloroform, Dibromochloromethane and Dichlorobromomethane as a total THMs due to direct and indirect intake of water. Bromoform component of THM was not considered due to no deduction from drinking water. Exposure and risk assessment was carried out by IRIS Integrated Risk Information System (USEPA, 2007) based on THMs exposure from oral ingestion, dermal adsorption and inhalation exposure. Present study considered average direct water intake as per Chowdhary study for risk evaluation from arsenic contamination in drinking water of the West Bengal area [22]. Consumption pattern was made as per Indian literature for peak risk assessment specifically closed to study area, on the basis that the ingestion rate was considered 4 & 3 L/day per person for adult males and females, respectively. While average life expectancy, body weight & body surface area for male and female were considered 64 & 67 years, 51.9 & 45.4 Kg and 1.54 & 1.38 m^2^ respectively [23,24]. Input parameter for risk exposure was considered as per Table 1.

Human health risk analysis for measuring concentration of THMs concentration was carried out by Integrated Risk Information System [25] & Risk Assessment Information System [26] with input parameters for the human risk analysis as summarized in Table 1. Following equations are used to evaluate the risk analysis.

(i) $$\mathrm{CD}I_{\mathrm{Ingestion}}\left(\mathrm{mg}/\mathrm{kg}-\mathrm{day}\right)=\frac{\mathrm{Cw}*\mathrm{IR}*\mathrm{EF}*\mathrm{ED}}{\mathrm{BW}*\mathrm{AT}}$$

(ii) $$\mathrm{CD}{I_{\mathrm{Dermal}}}_{\mathrm{absortion}}\left(\mathrm{mg}/\mathrm{kg}-\mathrm{day}\right)=\frac{\mathrm{Cw}*\mathrm{SA}*F*\mathrm{PC}*\mathrm{ET}*\mathrm{EF}*\mathrm{ED}}{\mathrm{BW}*\mathrm{AT}}$$

(iii) $$\mathrm{CD}I_{\mathrm{Inhalation}}\left(\mathrm{mg}/\mathrm{kg}-\mathrm{day}\right)=\frac{\mathrm{Cair}*\mathrm{VR}*\mathrm{AE}*\mathrm{ET}*\mathrm{EF}*\mathrm{ED}}{\mathrm{BW}*\mathrm{AT}}$$

**Table 1:** Input parameter for risk analysis

**S. No.**	**Input parameter**	**Units**	**Values**	**References**
1.	THMs Concentration in drinking water (Cw)	mg/L	0.357 to 0.594	As per analysis in present study
	(Maximum Concentration of THMs considered for each treatment plant for whole season)			
2.	THMs Concentration in air	mg/L	Little’s model	[16]
3.	Inhalation rate (IR)	m^3^/h	Male:0.84	[27]
			Female:0.66	
4.	Ingestion Rate (IR)	L/day	Male: 04	[22]
			Female:03	
5.	Exposure Frequency (EF)	Events/year	365	[28]
6.	Exposure Duration (ED)	Year	Male: 64	[23]
			Female:67	
7.	Exposure Time (ET)	Min/day	35	[26]
8.	Ventilation rate (VR)	m^3^/h	0.83	[27]
9.	Absorption efficiency (AE)	%	100	Maximum Risk
10.	Bathroom volume (Vs)	m^3^	5	[16]
11.	Average Time (AT)	Days	Male: 64X365	[23]
			Female:67X365	
12.	Body weight (BW)	Kg	Male: 60	[24]
			Female:55	
13.	Permeability Coefficient (PC)	cm/h	a) 0.00683 (CHCL_3_)	[26]
			b) 0.00402 (CHCL_2_Br)	
			c) 0.00289 (CHCLBr_2_)	
			d) 0.00235 (CHBr_3_)	
14.	Fraction of skin in contact with water (F)	%	100	Maximum Risk
15.	Skin surface area (SA)	m^2^	Male: 1.66	As per body weight and height.
			Female:1.53	
16.	Water flow rate (Q_L_)	L/min	5	[16]
17.	Air flow rate (Q_G_)	L/min	50	[16]
18.	Water Temperature (T)	°C	40	[29]
19.	Henry’s law constant (H)	Unit less	a) 0.25 (CHCL_3_)	[16,26,29]
			b) 0.124 (CHCL_2_Br)	
			c) 0.0526 (CHCLBr_2_)	
			d) 0.0501 (CHBr_3_)	
20.	Mass transfer coefficient (K_OL_ A) ^a^	L/min	a) 7.4 (CHCL_3_)	[16]
			b) 5.9 (CHCL_2_Br)	
			c) 4.6 (CHCLBr_2_)	
			d) 3.7 (CHBr_3_)	

Cair was calculated by following equations proposed by Little theory in terms of concentration of THMs in the bathroom;

$$\mathrm{Cair}=\frac{\mathrm{Ys}\left(t\right)+\mathrm{Ys}\left(i\right)}{2}$$;

Whereas

Ys(i) is the initial THM concentration in the shower room (assumed as 0 mg/L),

Ys(t) is the THM concentration in the shower room at time t (min).

Ys(t) = [1-exp (−bt)] (a/b); Whereas

$$a=\frac{\mathrm{QL}*\mathrm{Cw}\left\{1-\exp\left(-N\right)\right\}}{\mathrm{Vs}}$$

$$b=\frac{\left[\frac{\mathrm{QL}}{H}*\left\{1-\exp\left(-N\right)\right\}\right]+\mathrm{QG}}{\mathrm{Vs}}$$;

Whereas

N = (K_OL_ A)/Q_L_; Whereas N is a dimensionless coefficient,

Q_L_ = Water flow rate in liter per minute; a = factor; b = factor; t = time of contact.

Total Risk = (CDI _Ingestion_ X CSF_Oral_) + (CDI _Dermal_ X CSF_Dermal_) + (CDI _Inhalation_) X CSF _Inhalation_);

Where CDI is chronic daily intake and CSF is a carcinogenic slope factor.

Human health risk analysis calculated by the above formula in reference to potential cancer risk to the human body under existing conditions. Cancer slope factor (CSF) for oral, dermal and inhalation was taken from the standard value developed by RAIS and IRIS as given in Table 2.

**Table 2:** Cancer slope factor (CSF) for THMs

**S. No.**	**THMs**	**Carcinog- enicity**	**CSF Value (mg/kg-day) **^**−1**^		
			**Oral**	**Dermal**	**Inhalation**
1.	Chloroform (CHCL3)	Probable	6.10*10^-3^(RAIS)		8.40*10^-2^(RAIS)
2.	BDCM (CHCl2Br)	Probable	6.20*10^-2^ (IRIS)		
3.	DBCM (CHClBr2)	Possible	8.40*10^-2^(IRIS)		
4.	Bromoform (CHBr3)	Probable	7.90*10^-3^(IRIS)		3.90*10^-3^(RAIS)

Oral, dermal and inhalation CSF for BDCM, DBCM and oral and dermal CSF for chloroform and bromoform is used commonly as per a study [30,31].

According to USEPA method THMs exposure to the body was assessed from the equations as discussed in material and methodology section. In addition, USEPA has recommended various standard values for risk evaluation from THMs in drinking water [28]. Worldwide researchers carried out the risk assessment through standard guideline developed by the regulatory authority of USEPA, while many authors incorporated exposure duration, body weight, bathroom volume and skin surface area as per life expectancy of their country [29,32,33].

### Sensitivity analysis

Sensitivity analysis was performed by a radar chart, also known as a spider chart or a star chart because of its appearance, plots the values of each category along with a separate axis. Data were arranged in columns and rows in an excel worksheet with allowing all input parameter which is responsible for cancer risk as well as value of cancer risk from each route.

## Result and discussion

Risk estimation was calibrated from the maximum concentration of THMs considered for each treatment plant for the whole season. THMs value given in Table 3 as a source of drinking water for nearby areas of specific water treatment plant.

**Table 3:** Average THMs value and potential cancer risk for selected WTP

**S.N.**	**Water Treatment Plant**	**THMs Conc. (mg/L)**	**Male Cancer Risk**	**Female Cancer Risk**
1.	Subernrekha WTP, Ranchi	0.357	7.35E-04	7.11E-04
2.	ADDA WTP, Durgapur	0.362	6.53E-04	6.37E-04
3.	IGWTP, Kolkata	0.523	9.18E-04	9.18E-04
4.	MAITHON WTP, Maithon	0.536	9.37E-04	9.41E-04
5.	MADA WTP, Dhanbad	0.569	9.38E-04	9.42E-04
6.	BTPS WTP, Bokaro	0.594	1.13E-03	1.12E-03
7.	CTPS WTP, Chandrapura	0.566	1.08E-03	1.08E-03
8.	TISCO WTP, Jamadoba	0.413	8.04E-04	7.96E-04
Average Cancer risk Value of selected treatment			8.99E-04	8.92E-04

### Ingestion route evaluations of lifetime cancer risk for THMs

**Figure 2:**
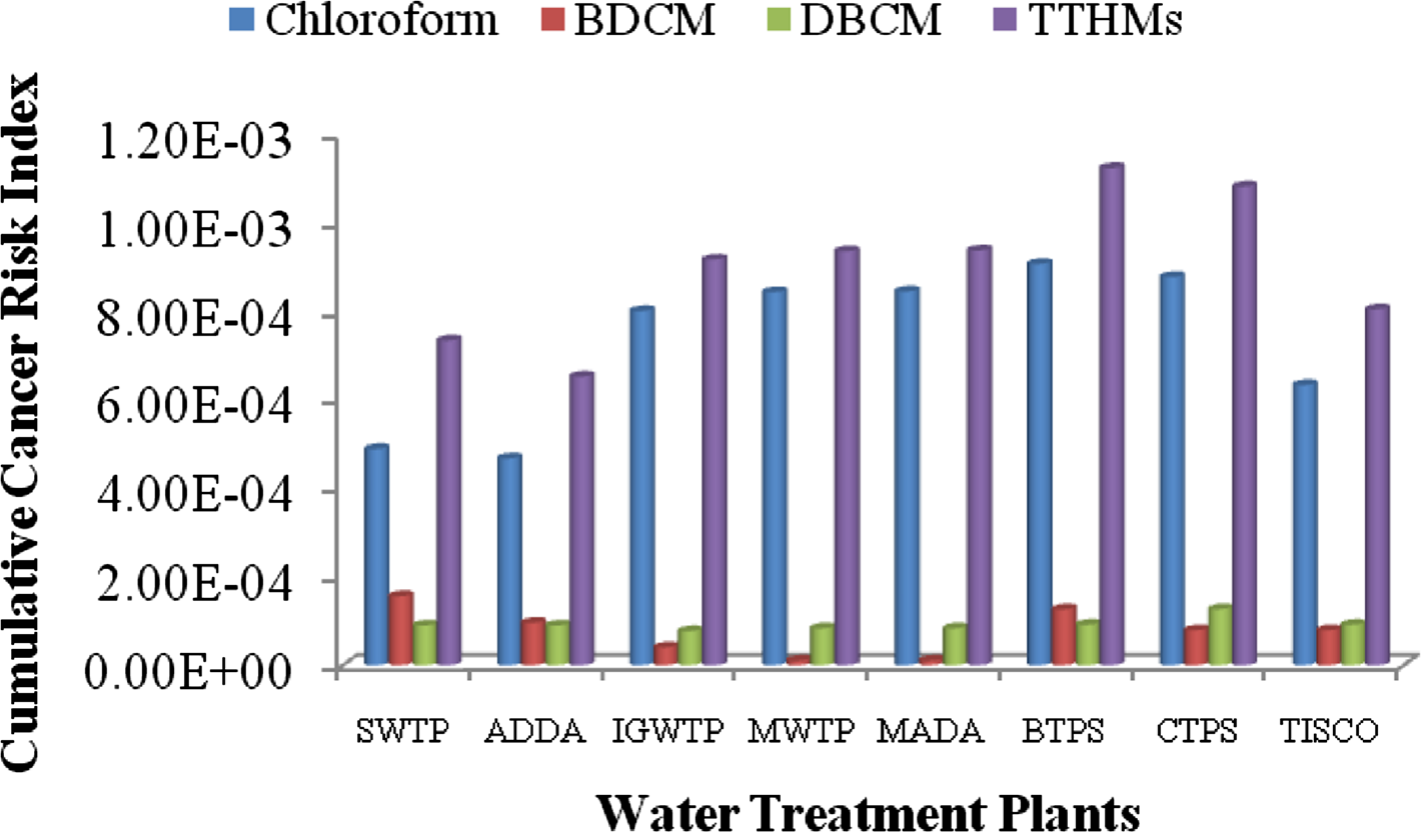
Route cancer risk for males from THMs in the drinking water.

**Figure 3:**
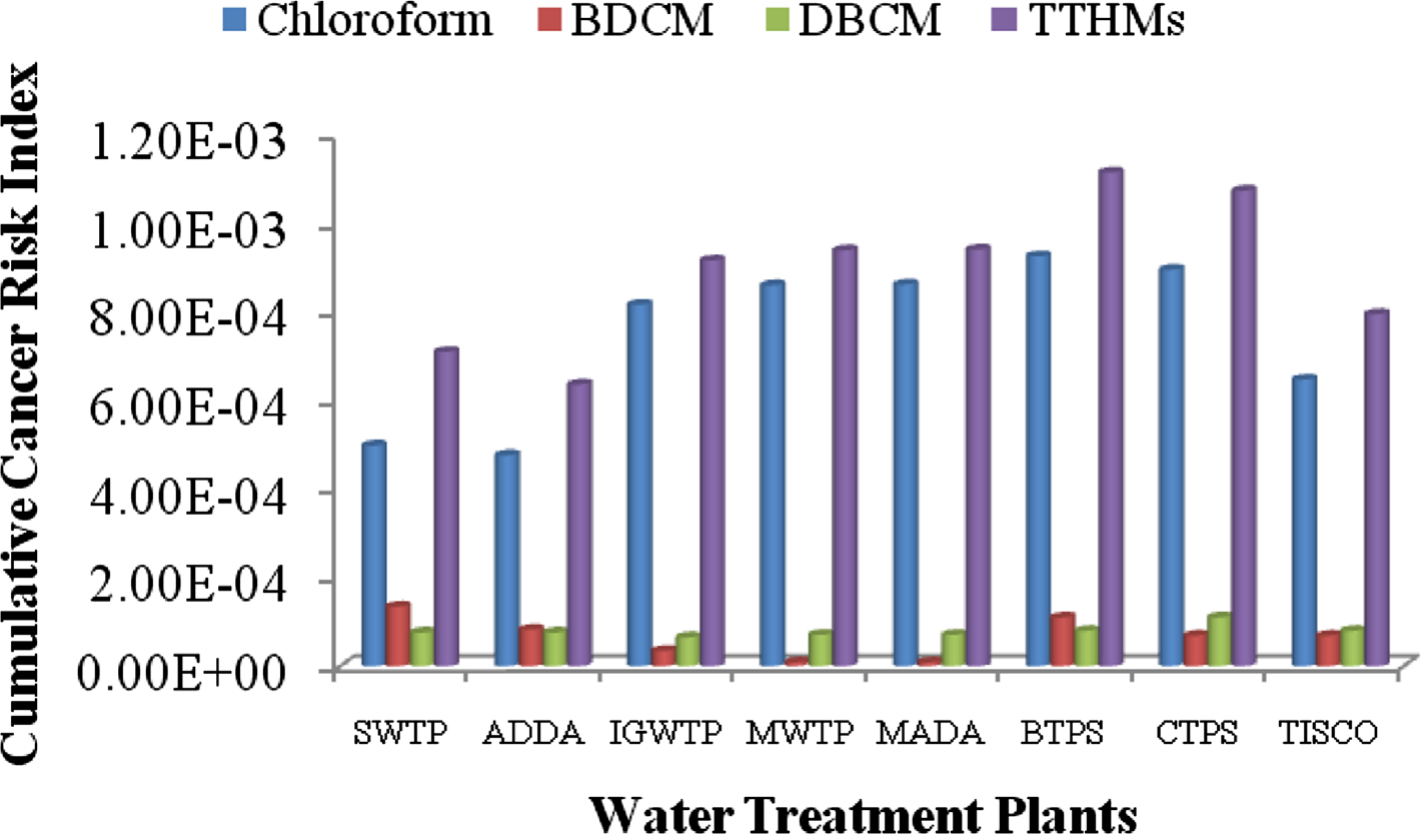
Route cancer risk for females from THMs in the drinking water.

The potency factor of the all THMs that are linked up with lifetime cancer risk for the exposed individuals is changing from individual THMs. The cumulative cancer risk through ingestion route for THMs were found 3.18E-04 and 2.60E-04 for male and female respectively, which is 318 and 260 times more than the prescribed limit given by USEPA. The cancer risk through oral consumption is shown in Figures 2 and 3 for male and female respectively. The lifetime cancer risks from Chloroform was found maximum in supply water, which was found 1.81E-04 and 1.48E-04 for male and female respectively, which is 56% of total contributor in cancer risk through the ingestion system. This higher risk factor from chloroform revealed that the concentration of chloroform is much higher than the prescribed limit as per IS 10500 and USEPA. Researches also reported that chloroform made the highest percentage contribution to total risks [34,35].

The average human lifetime cancer risk for BDCM was found 5.99E-05 and 4.90E-05 for male and female respectively in supply water of all water treatment plants which is 59.9 and 49 times greater than the permissible limit proposed by USEPA. Whereas in two water treatment plants, namely MWTP and MADA were shown the same cancer risk for BDCM as prescribed by USEPA.

The lifetime cancer risk for DBCM was found 7.70E-05 and 6.30E-05 for male and female respectively in supply water of all water treatment plants which is 77 and 63 times greater than the permissible limit proposed by USEPA. Higher risk of DBCM over BDCM was pertain even when the concentration of DBCM was found less than DBCM. The outcome revealed that due to higher slope factor DBCM shows more dominant in cancer risk.

The average lifetime cancer risk for THMs was found much higher than USEPA limit. The lifetime cancer risks for total THMs in all water treatment plants were higher than 10^−4^. Overall, the average lifetime cancer risk for total THMs through ingestion route in all water supply water is 3.18 × 10^−4^ and 2.60 × 10^−4^ which was higher than the USEPA acceptable risk by about 318 and 260 times for male and female respectively. Higher risk revealed that THMs concentration in supply water is more lofty than the USEPA prescribed limit. Risk estimation for female was found less which implies that the water consumption frequency reduces the chronic daily intake capacity in such concentration even when the female has a higher exposure duration compared to men.

### Dermal pathway evaluations of lifetime cancer risk for THMs

Polluted water can penetration the contaminants into the body by dermal pathway. Showering, bathing, laundry and swimming can be contamination contributors for risk exposure in the form of carciogenically. Dermal pathway for TTHMs is directly dependent upon the skin surface area, whereas USEPA given an average value for skin-surface area for male and female (male = 1. 94 m^2^, female = 1. 69 m^2^) as per height and other parameter for US population.

The cancer risk of THMs through dermal absorption exposure for both males and females are presented separately in Figures 2 and 3. Here, the values of potency factors for oral route are used to calculate the cancer risks of THMs through dermal contact as per the prescribed method given by RAIS, 2009. The lifetime cancer risks of Chloroform, BDCM and DBCM through dermal contact from supply water for males in almost all water treatment plants were found in average 1.81E-05, 3.50E-06 and 3.23 E-06 respectively. While the average cancer risk for females through dermal pathway were found 1.81E-05, 3.52E-06 and 3.25E-06 for Chloroform, BDCM and DBCM.

Among the all THMs, Chloroform had the highest lifetime cancer risk, followed by DBCM and BDCM for males and females. Overall, the average lifetime cancer risk for total THMs through the dermal pathway in supply water is 2.47E-05 and 2.48E-05 for male and female respectively, which was higher than the USEPA acceptable risk by approximately 24.7 and 24.8 times for male and female respectively.

### Inhalation pathway evaluations of lifetime cancer risk for THMs

Inhalation exposure occurs when the air breathed contains compounds volatilized during water usage, such as bathing, showering, laundering, and cooking. Showering has been identified as the activity contributing the greatest amount to inhalation exposure to volatile compounds [36]. Due to its property of a lower boiling point, chloroform is assumed to be the major compound to which people are exposed during showering and bathing. As a result, the cancer risk of THMs through inhalation from chloroform compound play important role in present study. Concentration of THMs in the air was calculated by Little theory in terms of concentration of THMs in the source water [16]. The results of cancer risk through Inhalation pathway are indicated in Figures 2 and 3.

In all water treatment plants chloroform was found in the range of 90 to 95% of total THMs rather than BDCM and DBCM which poses a higher cancer risk by inhalation pathway. Among the all THMs, Chloroform had the highest lifetime cancer risk, followed by DBCM and BDCM for males and females. Overall, the average lifetime cancer risk for total THMs through the inhalation pathway in all water supply water is 5.57E-04 and 6.07E-04 which was much higher than the USEPA acceptable risk by approximately 557 and 607 times for male and female respectively. Risk multiplication for female was found high due to high chronic intake of TTHMs through inhalation pathway which implies that high exposure duration increases the chronic daily intake of THMs from inhalation pathway. The lifetime cancer risks from Chloroform was found maximum in supply water, which was found 5.34E-04 and 5.83E-04 for male and female respectively, which is 95.8% of total contributor in cancer risk through inhalation system.

The lifetime cancer risks of BDCM and DBCM through inhalation from supplying water for males in all water treatment plants were found in average 1.11E-05 and 1.13E-05 respectively. While the average cancer risk for females were found 1.22E-05 and 1.23E-05 for BDCM and DBCM.

### Multipathway evaluations of lifetime cancer risk for THMs

**Figure 4:**
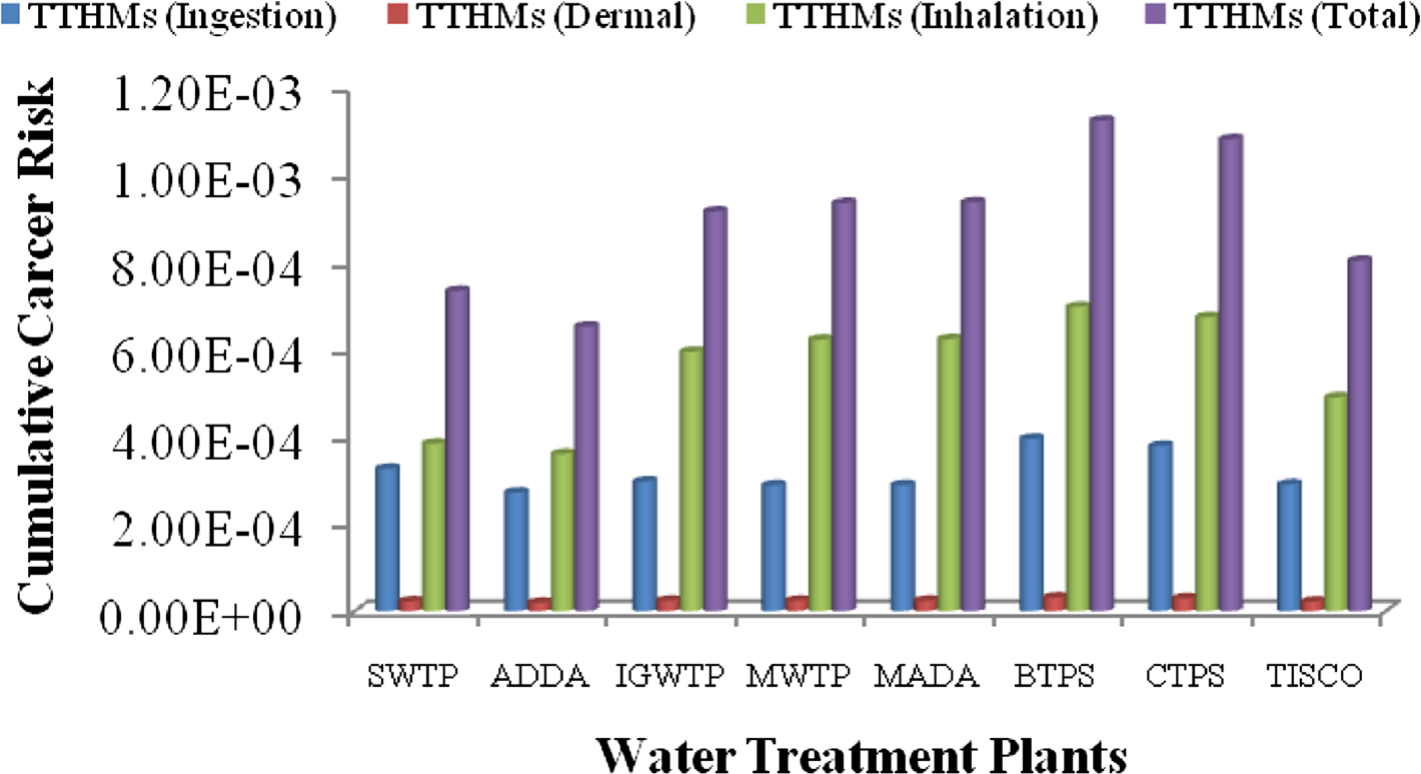
Multiple pathway cancer risk for males from THMs in the drinking water.

**Figure 5:**
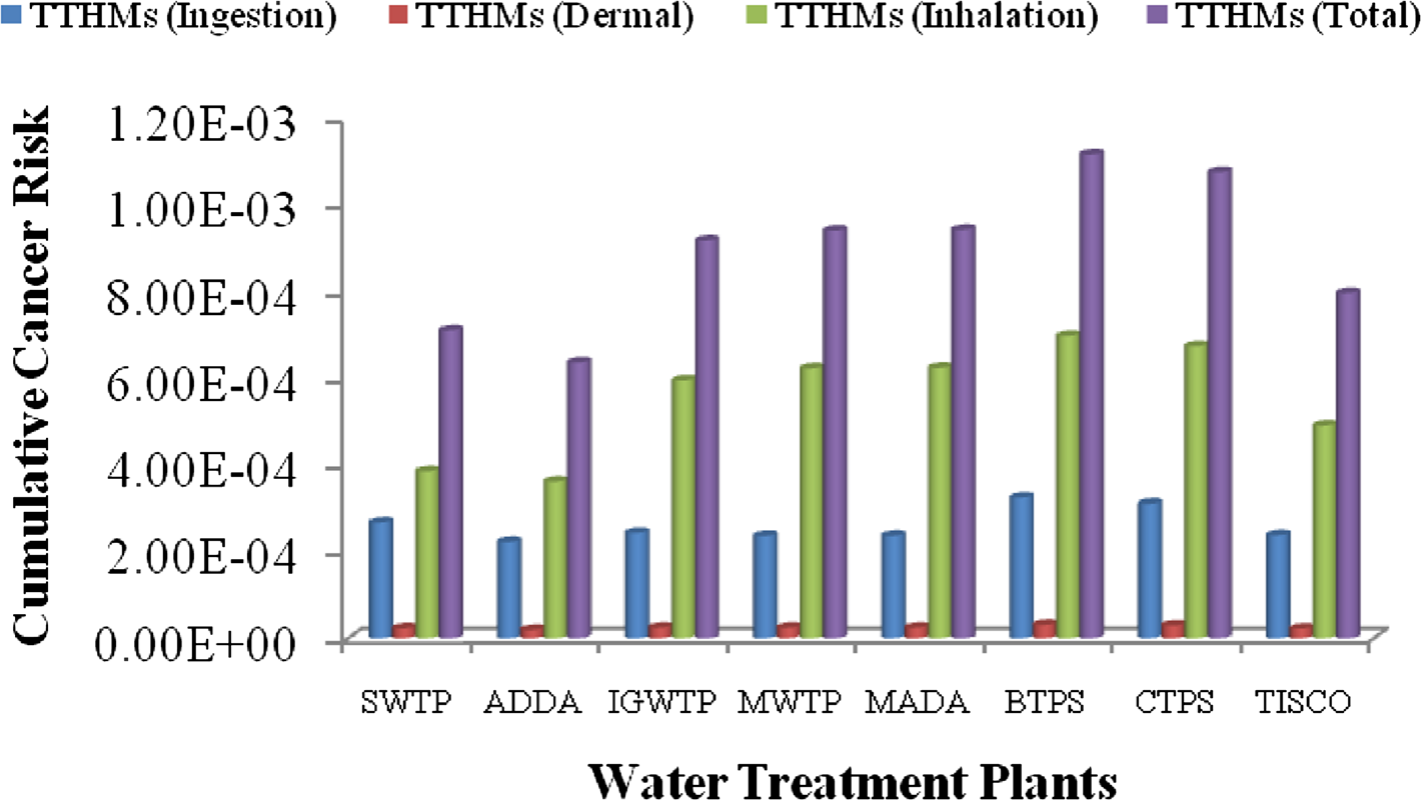
Multiple pathway cancer risk for females from THMs in the drinking water.

The multipathway evaluations of lifetime cancer risks for THMs exposure through ingestion, dermal absorption, and inhalation exposure were examined at the highest degree of danger. In these estimations, body weight was taken as 60 and 55 kg for male and female, respectively as per the Indian condition [24]. The median life span for males in India is 64, while that for females is 67 years [23]. The average water ingestion rate considered for oral cancer risk calculations was 4.0 and 3.0 L/day for male and female [22]. In inhalation risk calculations, the daily dose was calculated by putting on 5 m^3^ as per the most common Indian condition. The chloroform concentration in air used for the approximation of risk through inhalation was calculated using a Little model for THMs concentration as per bath-room volume. The results of multi-pathway cancer risk evaluation of water supply are shown in Figures 2 and 3 for male and female residents of eastern part of India, respectively. Figures 4 and 5 depict that the major cancer risk for both male and female residents is by chloroform through the inhalation pathway. The cancer risk for the all water treatment plants exceeded the acceptable level by a factor of 24.7 to 607 for both sexes. When the average lifetime multiway cancer risks for each THM in all water treatment plants were estimated, it appeared that the average risk due to chloroform was the highest among the all THMs. On the other hand, while there was no bromoform and therefore no associated risk in the supply water were considered.

### Sensitivity analysis of cancer risk

**Figure 6:**
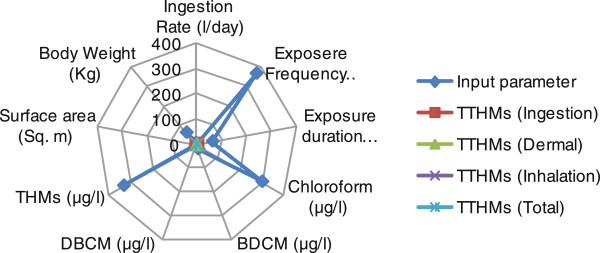
Radar plots for sensitivity analysis for cancer risk in males for drinking water.

**Figure 7:**
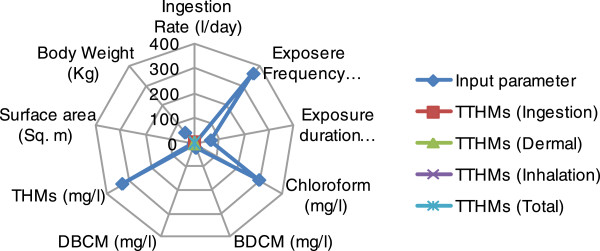
Radar plots for sensitivity analysis for cancer risk in females for drinking water.

Sensitivity analysis for risk potential were carried by the radar sensitivity tool for all water treatment plants as easily as for paths of THMs route for male and female both. Sensitivity analysis indicates the dominant factor for cancer risk in each pathway. Figures 6 and 7 shows that the risk rating from each route were centrally optimized by THMs concentration with special reference to chloroform concentration and exposure frequency. Body weight and exposure duration also show their little but strong sensitivity for risk valuation. The trends of sensitivity analysis were found same for males and females for all water treatment plants.

## Conclusion

This work concentrated on the assessment of the Human health risk assessment, exposure to THMs during drinking and bathing purpose as a cancer risk through the route of ingestion, dermal contacts and inhalation. The risk assessment models adopted in this study were developed by the USEPA and IRIS have been widely used in the risk assessment. The existing database in the IRIS provides a simple and reliable means of evaluating lifetime cancer risks for any population with dataset like THMs concentration, ingestion rate, body weight, life time and skin area for respective population and condition. Present study considered ingestion rate, body weight, life time and skin area as per Indian population with the maximum chance of absorption efficiency and fraction of skin contact to get maximum chance of risk from any route of transformation. The concentration of total THMs in selected water treatment plants was found in the range of 269–594 μg/L. Which is a lot higher than MCLs of the USEPAStandard phase I (80 μg/L) & phase II (40 μg/L). The value of individual THMs like chloroform is higher than USEPA, WHO and Indian standard IS 10500. Thacker also reported THM concentration in Mumbai city up to 337.5 μg/L, he also pointed the maximum concentration of chloroform in THM compound, but the authors did not predict any cancer risk for their study area [37]. The total cancer risk reached 8.99E-04 and 8.92E-04 for males and females, respectively, the highest risk from THMs seems to be from the inhalation route followed by ingestion and dermal contacts. [38-40] also reported the risk from THMs is transferred by the inhalation route followed by ingestion and dermal contacts. It reveals that water containing THMs of the selected water treatment plant of the eastern part of India was unsafe in terms of risk evaluation through inhalation and ingestion, while dermal route of risk was found very close to permissible limit of USEPA.

When the average lifetime multipath way cancer risks for each THM in drinking water were estimated, it appeared that the average risk due to chloroform was the highest among the three THMs. On the other hand, while there was no bromoform and therefore no associated risk in the drinking water due to bromoform, which revealed that the cancer risk for THMs was, in descending order, chloroform, dichlorobromomethane, and dibromochloromethane for both males and females. Sensitivity analysis shows that every input parameter is sole responsible for total risk potential, whereas exposure duration playing important role for estimation of total risk potential.

## Competing interests

The authors declare that they have no competing interests.

## Authors' contributions

This work is part of the Ph. D thesis of corresponding author, whereas both co-authors enhance the quality of paper by interpreting the result for Indian condition. All authors read and approved the final manuscript.
